# The Application of Carbon Nanomaterials in Sensing, Imaging, Drug Delivery and Therapy for Gynecologic Cancers: An Overview

**DOI:** 10.3390/molecules27144465

**Published:** 2022-07-13

**Authors:** Changji Xiao, Changming Li, Jun Hu, Lirong Zhu

**Affiliations:** 1 Obstetrics and Gynaecology Department, Peking University First Hospital, Peking University, Beijing 100034, China; xcji1988@163.com; 2 State Environmental Protection Key Laboratory of Food Chain Pollution Control, School of Ecology and Environment, Beijing Technology and Business University, Beijing 100048, China; lichangming@btbu.edu.cn

**Keywords:** gynecologic cancers, carbon nanomaterials, diagnosis, therapy, clinical application

## Abstract

Gynecologic cancers are one of the main health concerns of women throughout the world, and the early diagnosis and effective therapy of gynecologic cancers will be particularly important for the survival of female patients. As a current hotspot, carbon nanomaterials have attracted tremendous interest in tumor theranostics, and their application in gynecologic cancers has also been developed rapidly with great achievements in recent years. This Overview Article summarizes the latest progress in the application of diverse carbon nanomaterials (e.g., graphenes, carbon nanotubes, mesoporous carbon, carbon dots, etc.) and their derivatives in the sensing, imaging, drug delivery, and therapy of different gynecologic cancers. Important research contributions are highlighted in terms of the relationships among the fabrication strategies, architectural features, and action mechanisms for the diagnosis and therapy of gynecologic cancers. The current challenges and future strategies are discussed from the viewpoint of the real clinical application of carbon-based nanomedicines in gynecologic cancers. It is anticipated that this review will attract more attention toward the development and application of carbon nanomaterials for the theranostics of gynecologic cancers.

## 1. Introduction

Gynecologic cancers are one of the main health concerns of women throughout the world. They are a group of any malignancies affecting the reproductive tissues and organs of women including ovaries, uterine, cervix, vagina, vulva, and endometrium [1,2,3]. Cervical cancer, ovarian cancer, and endometrial cancer can account for over 10% of all deaths from cancer among females [4,5]. The early diagnosis and timely treatment of gynecologic cancers in women can effectively prevent the spread of cancer cells and significantly increase the 5-year survival rate of female patients [6]. Unfortunately, many female patients with gynecologic cancers are in mid or even late stage due to poor screening techniques and non-specific symptoms [7]. In the area of cancer therapy, the aggressive treatment for these cancers by traditional surgery, chemotherapy, and radiation can indeed acquire improved prognosis and survival quality [8,9], but still face a lot of difficulties such as neoplasm recurrence, insufficient treatment monitoring, limited therapeutic responses, intolerable cytotoxicity, and multiple drug resistance [10,11]. Therefore, the development of advanced diagnosis and therapy technologies is still a long-term goal in the field.

Nanomaterials when engineered together with biotechnology open a fascinating field in the diagnosis and treatment of diverse cancers [12,13,14,15,16]. Carbon nanomaterials such as graphenes, carbon nanotubes, fullerenes, carbon nanoparticles, nanodiamonds, carbon nanohorns, and carbon dots have attracted tremendous interest for their potential applications in tumor theranostics benefiting from their π-unique electron cloud and structures [17,18,19,20]. The most attractive properties of carbon nanomaterials for biological applications are the broad absorption of light in the UV–Vis–NIR region, NIR photoluminescence, unique Raman signals, exceptional photothermal response, photosensitized production of singlet oxygen, and large surface area for the covalent and non-covalent conjugation of contrast agents and drugs/DNA/RNA [21,22,23]. In recent years, carbon nanomaterials have been booming in the theranostics of malignant tumors including sensing, imaging, drug delivery, and photothermal therapy [24]. In particular, the application of carbon nanomaterials in gynecologic cancers has also been developing rapidly with great achievements, and some carbon nanomaterials even have been successfully applied to clinical practices such as carbon nanoparticle suspension injection [25]. As a current hotspot, the systematic summarization, generalization, and analysis of the recent advances in the application of carbon nanomaterials in gynecologic cancers are very necessary to stimulate the further development of the field.

In recent years, great success has been achieved in the application of various carbon nanomaterials in different gynecologic cancers including ovarian, cervical, and endometrial cancers. Although many overviews have summarized the progress of carbon-based materials as the nano platform for the detection and therapy of cancer-related diseases [24,26,27,28,29,30,31,32], few reviews have paid attention to the achievements in the theranostics of gynecologic cancers with carbon-based nanomedicines. In view of the rapid development and progress in this field in the past five years, this review comprehensively summarizes the latest progress in the application of various carbon nanomaterials (e.g., mesoporous carbon, graphenes/nanotubes, carbon nanoparticles, carbon dots, etc.) and their derivatives in the theranostics of different gynecologic cancers including ovarian, cervical, and endometrial cancers. Emphasis is mainly placed on reviewing the unique functionality of carbon-based nanomaterials in sensing, imaging, drug delivery, and therapy for gynecologic cancers. The advantages and potential problems of gynecologic cancers theranostics with these carbon-based nanomedicines are introduced in detail. The challenges and prospects in the future development of this field are also discussed and proposed in terms of the real clinical application. It is anticipated that this *Overview Article* will attract more attention toward the application of carbon nanomaterials in gynecologic cancers and encourage future clinical studies to push forward the advancement of this exciting area.

## 2. Sensing

The diagnosis of cancer in the early stages is vital for increasing the survival rate and succeeding in the treatment in a cost- and time-effective manner [33,34]. Many cancerous processes in various tissues and organs are always accompanied by a change in biosignals including antigen, protein, redox state, biomolecules, genes, pH, or cancer cells themselves [26,35]. Sensing is a convenient technology to directly/indirectly detect the biosignals of cancers in vitro without trauma and expensive cost in common with a detailed physical examination. The sensing of cancer signals can contribute to the explanation of intricate biological processes and the development of advanced diagnoses [26]. The common biosignals for diagnosis of gynecologic cancers include cancer antigen 125 (CA 125), human epididymis protein 4 (HE4), H_2_O_2_ released by live cervical cancer cells, DNA point mutation, pH, and so on [36]. Carbon nanomaterials have great advantages in adjustable surface structures, good biocompatibility, and nontoxicity, and great achievements have been made for sensing gynecologic cancers with carbon nanomaterials in recent years.

**Figure 1:**
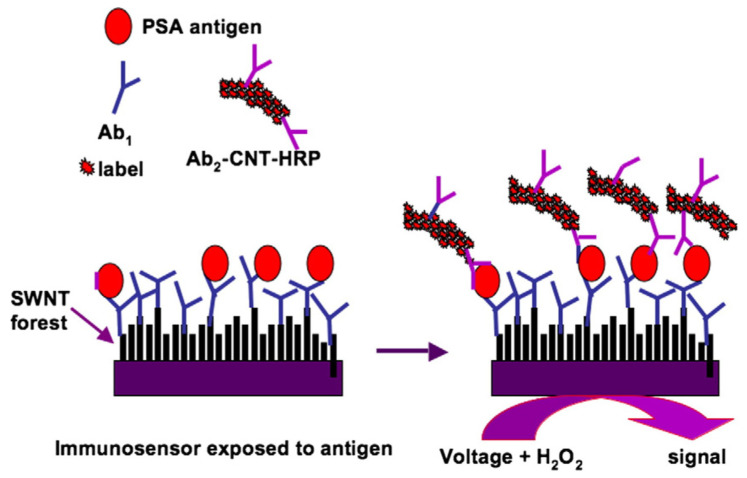
The typical fabrication strategy of bioelectronic immunosensors with carbon nanomaterials [41]. Copyright 2009, Elsevier.

Graphenes and carbon nanotubes are two typical carbon nanomaterials with unique physicochemical properties such as charge carrier mobility [37], thermal conductivity [38], and specific surface area [39,40], which have attracted enormous attention in biosensors. They are always used to modify the electrode as a platform with good conductivity, high specific surface area, as well as strong binding affinity for other nanoparticles and probe molecules to achieve high sensitivity of immunosensors, as shown in Figure 1 [41]. Moreover, the functionalization of graphenes and carbon nanotubes can further improve their conductivity, solubility, and biocompatibility [42,43]. Recent studies for the detection of CA 125 have shown that the modified electrodes by graphenes and carbon nanotubes always exhibit much better sensing performance owing to their higher binding affinity, conductivity, and specific surface area [33,44,45,46,47,48]. The graphene nanosheets can also increase the surface area and electrical conductivity of hierarchical nanohybrid microelectrode with enhanced electrocatalytic activity to track H_2_O_2_ secretion in human cervical cancer cells [49]. Taking advantage of the high charge transfer kinetics of graphenes, Tripathy et al. developed electrospun graphene-doped manganese III oxide nanofibers (GMnO) to detect single-point DNA mutations for early diagnosis of breast/ovarian cancer [40]. The presence of GMnO nanofibers on the bioelectrode surface leads to enhanced rate kinetics, thereby accounting for an improved resolution of the label-free electrochemical biosensor for BRCA1 gene-specific point mutation (185delAG) detection. Carbon-based nanohybrids can also detect normal fibroblasts and HeLa cancer cells through the surface-enhanced Raman scattering (SERS) ability by loading the noble metal nanoparticles as reported by Venkatakrishnan et al. [50]. They created a novel SERS-active nanoplasmonic-sensing platform based on a self-functionalized biocompatible 3D interconnected nanocarbon web (INW) structure, and the sub-10 nm physical morphology of the INW helps the endocytic uptake of INW clusters to cells with significantly enhanced factors of 3.66 × 10^4^ and 9.10 × 10^3^ for crystal violet and Rhodamine 6G dyes, respectively. These latest reports show that the graphenes and carbon nanotubes mainly play an indirect role with enhanced sensitivity and biocompatibility, but they have gradually become the basic and common auxiliary units in biosensors for the detection of various biosignals of gynecologic cancers.

**Figure 2:**
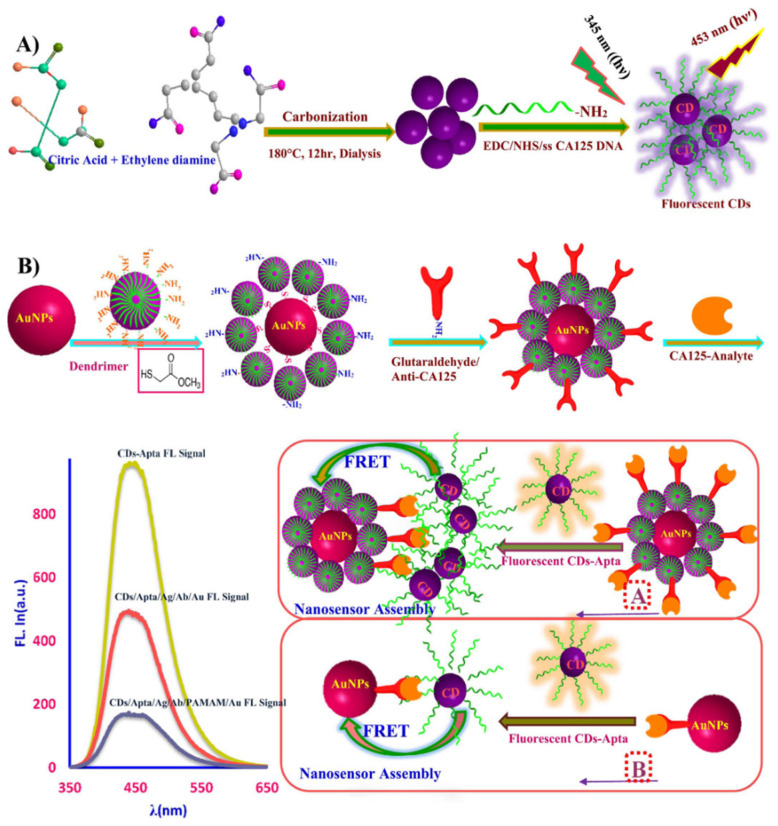
The mechanism for the FRET-based detection system: in the first part, the fluorescent CDs were conjugated to DNA (**A**); and then the CDs-Apta fluorescence response was quenched by AuNPs-PAMAM-Ab through FRET in the presence of CA125 analyte (**B**) [53]. Copyright 2017, Elsevier.

Carbon dots (CDs) are featured by a graphitic core mainly constituted by sp^2^ or sp^3^ carbon and size ranging from less than 20 nm to up 60 nm [28,51]. Different from the graphenes and carbon nanotubes, CDs with unique optical properties can directly work as a key component in biosensors to sense the various biosignals of gynecologic cancers. Luminescence resonance energy transfer-based probes show pronounced specificity and simplicity for CA125 detection, but they suffer from strong interference from autofluorescence of biosamples. To resolve this problem, CDs were utilized by Zhang et al. as energy acceptors for the overlap between the upconversion luminescence (UCL) spectrum of upconversion nanoparticles (UCNPs) and the absorption spectrum of CDs [52]. Aptamer-modified UCNPs were combined with CDs through π–π stacking interaction, and the formation of a CA125-aptamer complex blocked the π–π stacking and recovered the UCL, whose intensity increased linearly with the logarithm of the CA125 concentration in the range from 0.01 to 100 U mL^−1^. The probe shows great sensitivity to CA125 with a detection limit of 9 × 10^−3^ U mL^−1^. Hamd-Ghadareh et al. also reported a novel immunosensor for CA 125 cancer marker and OVCAR-3 cells based on a fluorescence resonance energy transfer from aptamer-CDs to an Ab-PAMAM-labeled Au nanoparticles (NPs) heterostructure, which was switched off by the specific interaction of CA125 Ag with aptamer and Ab [53]. Upon capping AuNPs, the intrinsic fluorescence of CDs becomes quenched and the addition of CA125 leads to a decrement in the fluorescence signal of CDs. The detection limits of CA125 Ag and OVCAR-3 cancer cells can reach 0.5 fg mL^−1^ at a wide concentration range up to six orders of magnitude and 4 cells/10 μL at a range of 2500–20,000 cells, respectively. The detailed detection mechanism can be seen in Figure 2. In the two typical cases, fluorescence resonance energy transfer signals between CDs and other nanoparticles play a key role in the fluorescence immunosensor to achieve the high sensitivity of CA125.

For other biological signals, Khan et al. fabricated a ZnO@CDs electrode with excellent sensing, and selective and reproducible performance for the detection of H_2_O_2_ released from HeLa cells. In addition to its high biocompatibility, CDs acted as an electric sink in the ZnO@CDs electrode with enhanced electrical conductivity and good separation efficiency of photo-induced electrons and holes, thus leading to improved photo-electro catalytic oxidation efficiency [54]. The nitrogen and sulfur dual-doped CDs reported by Gong et al. possessed an extraordinary emission escalation at an emission wavelength of 543 nm and displayed a favorable linear relationship in the physiological pH range of 5.0–7.4, which can be applied to real-time pH fluctuations monitoring in HeLa cells with negligible autofluorescence [55]. Han et al. also described nitrogen and sulfur dual-doped CDs with enhanced fluorescence by silver nanoparticles for the sensitive detection of the cancer biomarker human epididymis protein 4 (HE4) and ovarian cancer cells [34]. The fluorescent detection system demonstrated a highly sensitive and selective target-triggered “turn-on” fluorescence response toward HE4 over other proteins and common ions with a limit detection of 2.3 pM. According to the results of these CDs-based fluorescence sensors, the good fluorescent response and precise function relationships between the fluorescence intensity and targeted biosignals are of great importance in achieving a low detection limit.

The above latest research advances show that carbon nanomaterials (graphene, nanotubes, and CDs) have been extensively and successfully applied to the detection of various biosignals (e.g., CA 125, HE4, pH, H_2_O_2_, BRCA1 gene-specific point mutation, and cancer cells themselves) of gynecological cancers with a high sensitivity and low detection limit (Table 1). The novel design schemes of these biosensors with carbon nanomaterials were well demonstrated with a clear sensing mechanism of the detection procedure. The graphenes and carbon nanotubes with good conductivity, high specific surface area, and strong binding affinity have become the basic and common auxiliary units and function indirectly to improve the sensitivity of biosensors. CDs may possess sensitive fluorescent responses and can directly be used to detect the biosignals of gynecological cancer cells with a low detection limit. The physicochemical structures of these carbon-based nanomaterials are always well investigated to reveal their sensing mechanism. However, the indistinct biochemical environment with complex components in/out of the cancer cells may interfere with the accuracy and reliability of the biosensors. The different patients, ages, and other diseases may also affect the detected biochemical signals of gynecological cancers. Abundant clinical research is still necessary to estimate their application possibility in the real detection environment. Thus, the fabrication of practical and convenient devices based on this fundamental research is still greatly desired for their further clinical tests.

## 3. Imaging

Imaging is another promising technology to in vivo directly identify and distinguish the tumor sites precisely, which possesses the ability to monitor tumor morphologies in real time, allowing doctors to understand the evolution of tumor tissues and enabling the dosing of drugs to be adjusted to abate overtreatment of harmful side-effects, or undertreatment of incomplete cancer remission [56,57,58]. Carbon nanomaterials can be delivered in living cells or administered in vivo with or without bioconjugation. The broad absorption band extending in the UV–Vis–NIR region, NIR photoluminescence, excellent photoacoustic response, and unique Raman/SERS bands of some carbon nanomaterials, such as graphenes, carbon nanotubes, and CDs, may realize the spectral imaging of tumors cells [59,60]. On the other hand, the dark color of carbon nanoparticles may directly color-specific tissue with visible images by the naked eye and facilitate tumor-related surgery [61]. In recent years, imaging with carbon nanomaterials has developed rapidly in the theranostics of cancers. For gynecological cancers, the latest research mainly focuses on the photoluminescence bioimaging of cancers with CDs and the coloration of lymph nodes with carbon nanoparticle suspension injections.

**Figure 3:**
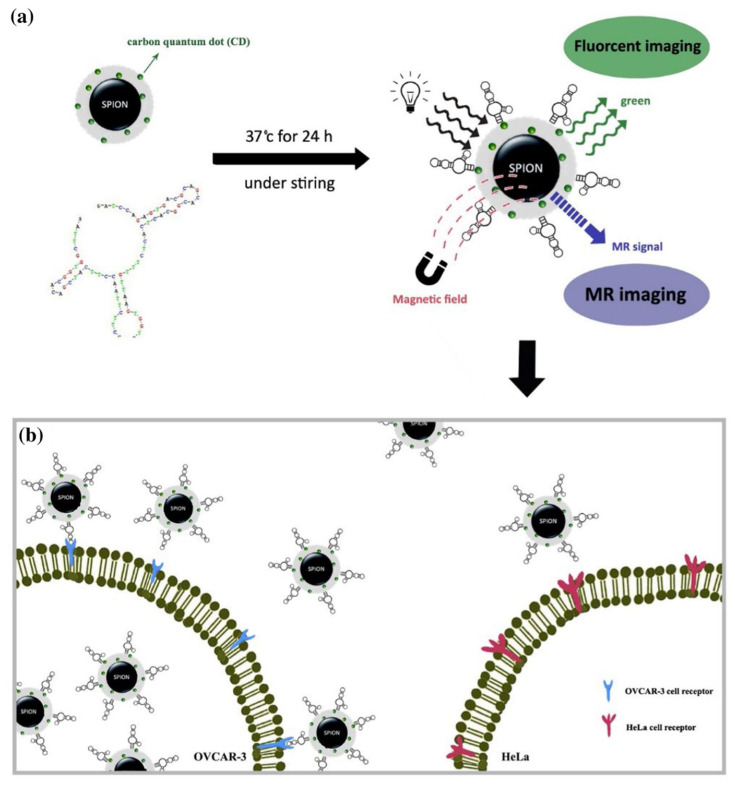
The illustration of the aptamer-targeted nanoprobe for multimodal fluorescence/magnetic resonance imaging (**a**) and different internalization of aptamer-superparamagnetic iron oxide nanoparticle-carbon dots into target and control cells (**b**) [65]. Copyright 2021, Springer.

With unique structural and optical properties, CDs and their derivatives have been applied to the photoluminescence imaging of various gynecological cancers. Besides sensing biological signals, the fluorescence of CDs-based nanomaterials can also realize the fluorescence labeling and quantitative determination of HE4-positive ovarian cancer cells [34], selective imaging of the OVCAR-3 line cells [53], and cellular multicolor imaging of Hela cells [62]. Recently, Zarghami et al. reported two nitrogen-doped green carbon dots (N-CDs) from lemon and tomato extraction in the presence of hydroxylamine with enhanced fluorescence efficiency, which enhanced fluorescence intensity and biocompatibility for bio labeling and bioimaging of Hela cells [63]. To overcome the easily diffuse and quench of CDs, connecting their functional groups with other nanoparticles such as hydroxyapatite (HAp) will obtain long-time fluorescence, as reported by Ma et al. [64]. They found that the CQD-HAp hybrid nanorods had prolonged fluorescence life due to the connection between CQDs and HAp nanorods, and possessed higher fluorescence quantum yield than pure CQDs with an improved fluorescence imaging effect for cervical cancer (Hela) cells. Coupling the CDs with magnetic nanoparticles can acquire multimodal fluorescence/magnetic resonance imaging of human ovarian cancer cells. Shokrani et al. introduced a multifunctional-aptamer nanoprobe consisting of TOV6 APT-superparamagnetic iron oxide nanoparticle-carbon dots (APTSPION-CDs) for fluorescence and magnetic resonance targeted imaging (FI/MRI) of human ovarian cancer cells (Figure 3) [65]. In vitro cellular uptake and signal enhancement of this multimodal FI/MRI nanoprobe demonstrated the potential application of APT-SPION-CDs as a contrast agent for MRI and as a fluorescent probe for fluorescence microscopic imaging. To further increase the biocompatibility, Filpponen et al. constructed biocompatible and photoluminescent nanohybrids comprising amino-functionalized carbon dots (NH_2_-CDs) and TEMPO-oxidized cellulose nanocrystals (TO-CNCs) via carbodiimide-assisted coupling chemistry, which was used as a bioimaging probe to investigate its interactions with HeLa and RAW 264.7 macrophage cells in vitro [66]. The surface conjugation with NH_2_-CDs not only improved the cytocompatibility of TO-CNCs, but also enhanced their cellular association and internalization on both HeLa and RAW 264.7 cells after 4 and 24 h incubation, as shown in Figure 3. These achievements manifest that the doping, modification, functionalization, and hybridization of CDs are always adopted to increase the fluorescence imaging intensity and stability, acquire multifunctional imaging, and reduce the biotoxicity.

Carbon nanoparticles suspension injection (CNSI), composed of carbon nanoparticle cores and polyvinylpyrrolidone K30 as the dispersion reagent, is the only commercialized carbon nanomaterial authorized for clinical application [67,68]. CNSI can migrate fast in lymphatic vessels and accumulate in lymph nodes, thus showing high performance in tumor drainage lymph node (TDLN) imaging by staining TDLN black after intratumoral injection [67,69]. CNSI has been successfully applied in surgical procedures on advanced gastric cancer, breast cancer, and papillary thyroid carcinoma [61,70,71,72]. Over 100,000 patients per year received CNSI during oncological surgery to recognize and eliminate the metastatic lymph nodes [67,73,74,75,76]. Experimental evaluations and clinical observations have collectively confirmed the biosafety of CNSI [67,77,78].

**Figure 4:**
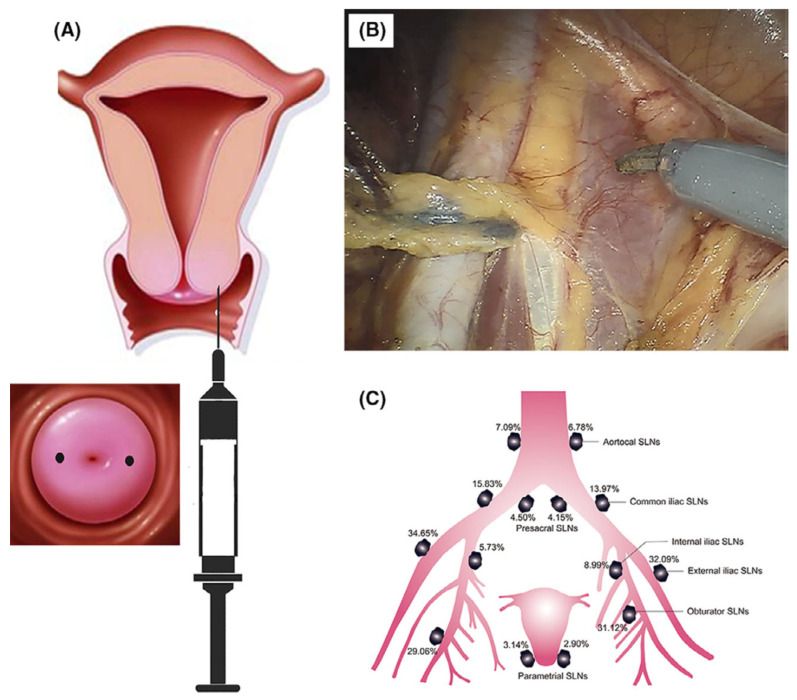
The technique of CNSI application in SLNB for assessing lymphatic spread of early-stage cervical cancer [81]. (**A**) Injecting CNSI into the ectocervix at the 3 and 9 o’clock positions superficially by a skin test needle, approximately 5–10 mm from the tumor border and perpendicular to the cervix surface. (**B**) The black-stained SLNB was identified adjacent to the external iliac artery and vein, which belonged to SLNB of external iliac region. (**C**) Locations of SLNBs on each side of the pelvis. Copyright 2020, Wiley.

For gynecological cancers, the metastatic lymph node ratio may be very low (e.g., only 9% for early endometrial carcinoma) [79], and the introduction of CNSI will be of great significance in reducing surgical range and injury via the imaging of metastatic lymph nodes by CNSI. In recent years, the clinical application effect of CNSI has also been investigated in the oncological surgery of gynecology. Wang et al. reported a case of 45 women with stage IB1-IIA1 cervical cancer who underwent sentinel lymph node biopsy (SLNB) mapping using CNSI during laparoscopic surgery [80]. The overall and bilateral detection rate was 93.3% (42/45) and 60.0% (27/45), respectively. The case survey indicates that laparoscopic SLNB mapping with CNSI may be simple and efficient for patients with early-stage cervical cancer. Ya et al. further carried out a prospective study of 356 cases to evaluate the clinical diagnostic validity of CNSI in SLNB for assessing the lymphatic spread of early-stage cervical cancer as shown in Figure 4 [81]. SLNB with CNSI had a sensitivity of 96.65%, a false-negative rate (FNR) of 4.35%, and a negative predictive value (NPV) of 99.29%, which demonstrates that SLNB with CNSI is safe, feasible, and relatively effective for guiding precise surgical treatment of early-stage cervical cancer.

For the application of CNSI in laparoscopic surgery of endometrial carcinoma, Zuo et al. executed a prospective consecutive study to evaluate the detection rate and accuracy of SLNB mapping using a cervical and fundal injection of CNSI in laparoscopic surgery of endometrioid endometrial cancer [82]. Fifty patients received fundal sub-serosal injections at four sites (fundal group), while 65 patients received cervical sub-mucosal injections at 2 sites (cervical group). SLNB mapping by CNSI in laparoscopic surgery for endometrial cancer is a safe and effective alternative with a higher detection rate and better accuracy in cervical injection than fundal injection. Recently, Chen et al. also conducted a retrospective study of seventy-six endometrial cancer patients who underwent SLNB mapping with or without systemic pelvic lymphadenectomy to explore the value of SLNB mapping with CNSI [83]. The overall and bilateral detection frequencies were 71.1% (54/76) and 61.1% (33/54), respectively. The detection frequency of SLNB mapping with CNSI in endometrial cancer patients was not as high as Zuo’s results and some other cancer types. More research is needed to find out the reason and improve SLNB mapping in endometrial cancer patients.

The above research advances manifest that carbon nanomaterials have promising application prospects in the imaging of gynecological cancers. Except for the in vitro cell imaging experiment, more animal tests and even clinical trials should be explored for the further application of carbon nanomaterials in gynecological cancers. As a typical success story, CNSI has been confirmed to be is safe, feasible, and relatively effective for guiding the precise surgical treatment of early-stage cervical and endometrial cancers. As the only commercialized carbon nanomaterials authorized in clinical application, CNSI could be immediately accessible for cancer patients through “off-label” use when developing new clinical applications. More stirring research findings will be anticipated for the wider application of CNSI in gynecological cancers with more powerful functions.

## 4. Drug Delivery

The large surface area, availability of multiple functional groups, and the hydrophilic/hydrophobic nature of carbon nanomaterials make them ideal host materials for the loading and delivery of various fluorophores, drugs, proteins, DNA, and siRNA [84,85]. The main functions of carbon nanomaterials for drug delivery include targeting vector, high local concentration, reduced side effects and drug resistance, and improved therapeutic efficiency. As reviewed above, carbon nanomaterials have gradually become the basic and common auxiliary units coupling with other nanoparticles and functional molecules for sensing and imaging gynecologic cancers [86]. This part will mainly focus on carbon nanomaterials as the gene/drug carrier for the therapy of gynecologic cancers.

**Figure 5:**
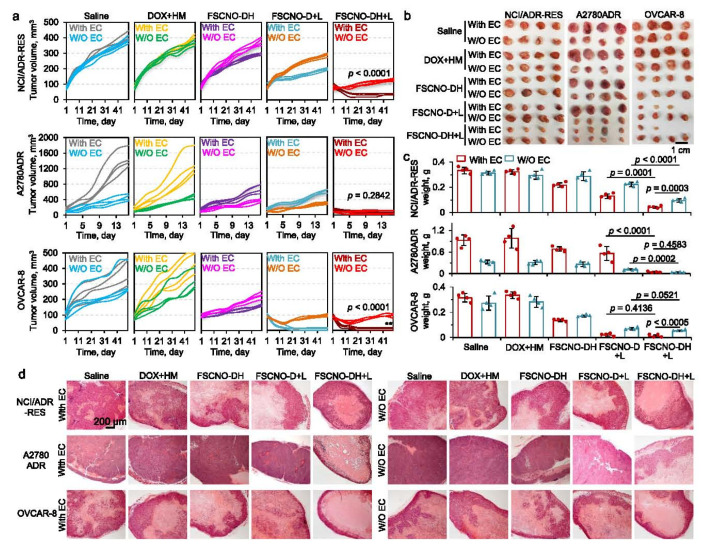
The capacity of the carbon nano-onion-based nanoparticles to overcome drug resistance and destroy drug-resistant tumors (NCI/ADR-RES, A2780ADR, and OVCAR-8) in vivo [92]. Growth curves (**a**), gross images (**b**), and weight (**c**) of NCI/ADR-RES, A2780ADR, and OVCAR-8 tumors with or without (W/O) co-injection of aHUVECs (With EC or W/OEC) in mice with various treatments, and representative images of hematoxylin and eosin (H&E) stained tumor tissues collected after sacrificing the mice on day 49 for the NCI/ADR-RES and OVCAR-8 groups and on day 16 for the A2780ADR group (**d**). Copyright 2021, Springer Nature.

Early studies found that carbon nanomaterials such as graphene oxides and mesoporous carbon nanoparticles could deliver membrane-impermeable chemical agents or genes into eukaryotic cells (HeLa) with good cellular uptake efficiency and biocompatibility [87,88]. Recently, graphene oxides coated with cationic lipids were reported as the carrier to deliver the double-stranded DNA into human cervical cancer cells (HeLa) and human embryonic kidney (HEK-293) cells [89]. Some genes such as siRNA supported on the carbon nanoparticles can be released upon NIR irradiation to realize the photoacoustic delivery of siRNA into ovarian cancer cells with a high concentration [90]. Wu et al. constructed a novel GSH-, pH-, and NIR-responsive targeted PGA-gatekeeper nanocarrier based on magnetic hollow and porous carbon-based nanoparticles for in vivo cancer therapy, and the as-prepared nanoparticles efficiently accumulated at tumor sites and inhibited the growth of tumor (HeLa) with minimal side effects [91]. The photoacoustic delivery ability of carbon nanoparticles can also overcome cancer drug resistance. Wang et al. invented carbon nano-onion-mediated dual targeting of P-selectin and P-glycoprotein to specifically release a P-gp inhibitor and anticancer drug into tumor cells [92]. The carbon nano-onions can exhibit superior light absorption properties in the near-infrared region after being decorated with TEOS and fucoidan, which can trigger drug release from the nanoparticle at a low NIR power, improve the bioavailability of anticancer drugs inside the cells (NCI/ADR-RES, A2780ADR, and OVCAR-8) and increase the systemic toxicity of a chemotherapy drug (Figure 5). For the uptake and delivery mechanism of carbon nanoparticles, Hifni et al. prepared fluorescently labeled carbon nanohorns to determine the factors influencing how internalization occurs and the destinations they reach in HeLa cells [93]. Carbon nanohorns were localized both at the cell periphery and in a juxtanuclear pattern inside HeLa cells through multiple mechanisms of endocytosis.

**Figure 6:**
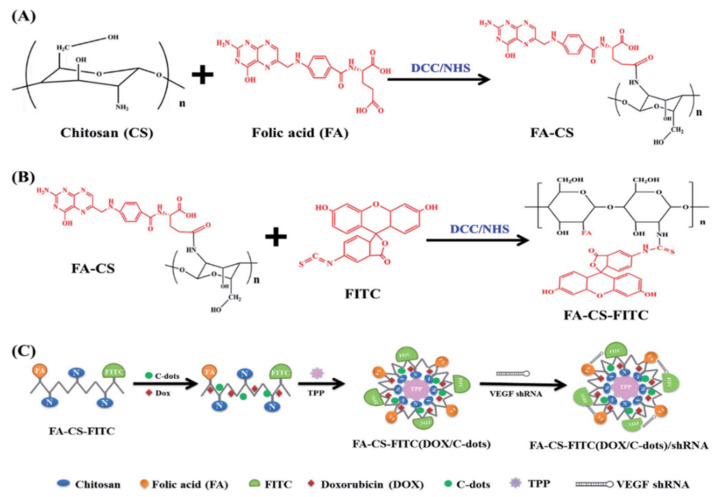
The illustration of the preparation and modification of FA–CS–FITC(DOX/C-dots)/VEGF shRNA nanoparticles [96]. Folic acid (FA) molecules were firstly conjugated to chitosan (CS) to form FA–CS (**A**), and then reacted with fluorescein isothiocyanate (FITC) to obtain FA–CS–FITC polymer complex (**B**). Doxorubicin (DOX) and carbon-quantum dots (C-dots) were doped, and VEGF shRNA was electrostatically absorbed on the nanoparticles (**C**). Copyright 2016, RSC.

CDs may possess high drug uptake with good biocompatibility (e.g., the loading of 28% doxorubicin (DOX) for glucose-derived CDs), which are also used as the drug carrier in the field of gynecologic cancers [94]. CDs with aspirin reported by Xu et al. have both anti-inflammatory and fluorescent biomarker functions, which can efficiently enter human cervical carcinomas and have effective anti-inflammatory effects with low cell toxicity in vitro and in vivo compared to aspirin only [95]. Liu et al. fabricated CD-based multifunctional FA–CS–FITC(DOX/C-dots)/VEGF shRNA nanocomplexes (Figure 6) including formic acid (FA) for the target, DOX as anticancer medicine, and fluorescein isothiocyanate (FITC) as a targeted drug/gene co-delivery nanovector for the image-guided and target-specific treatment of cancer [96]. The nanocomplexes demonstrated excellent dual fluorescence cellular imaging together with the enhanced synergistic antitumor activities for HeLa cells. Moreover, a magnetofluorescent nanohybrid comprising fluorescent CDs and magnetic iron oxide nanoparticles (IOs) with excellent colloidal stability was reported by Wen et al. as shown, which was used as the nanocarrier of a platinum-based drug with magnetically enhanced anticancer efficacy for HeLa cells both in vitro and in vivo [97]. It can be seen from these cases that the CDs are excellent drug carriers for genes, drugs, and other functional molecules, and the CD-based nanoplatforms always possess multiple functions including drug delivery, fluorescent biomarker, and therapy of the gynecological cancers.

In short, carbon nanomaterials including mesoporous carbon, graphene oxides, and CDs are excellent carriers for the delivery of genes, anticancer/anti-inflammatory drugs, and functional molecules into cervical and ovarian cancer cells. Coupling carbon nanomaterials with other nanoparticles as nanoplatforms for genes, drugs, and functional molecules can obtain pluri-potentiality (e.g., targeting delivery/release, fluorescence/magnetic marker, against drug resistance) for therapy of gynecological cancers. Although most studies claimed these carbon-based nanocarriers were well biocompatible with low toxicity, no clinical results have been reported till now. Thus, more clinical research is encouraged for the further application of carbon-based nanomaterials to deliver drugs for gynecological cancer cures.

## 5. Therapy

Therapeutic potentials of carbon nanomaterials in cancer therapy include using them as the drug delivery system, directly acting as anticancer drugs, and inducing photothermal therapy [85]. As discussed in the previous section, some anticancer drugs/genes such as DOX and siRNA can be loaded on the specific carbon nanomaterials via covalent or non-covalent force to form an efficient drug delivery system, which realizes the selective delivery of genes/drugs in cancer cells or the tumor milieu with enhanced therapeutic effect and minimized side effects [90,96]. The optical property of some carbon-based delivery systems (e.g., carbon nano-onion and CDs) can simultaneously possess optical imaging, chemical/photochemical-induced drug release, and drug-targeted therapy for gynecological cancers, as reviewed in the previous section [92,97].

Besides the drug delivery systems to facilitate cancer therapy, the carbon-based nanohybrids can be directly used as anticancer drugs for tumor inhibition. Gurunathan et al. developed the reduced graphene oxide–silver (rGO–Ag) nanocomposites using the *Tilia amurensis* plant, which significantly inhibited the viability of ovarian cancer cells compared with graphene oxide, rGO, and Ag NPs [98]. Recently, they further found that the combination of rGO-AgNPs and trichostatin A (TSA) can cause potential cytotoxicity and induce significantly greater cell death compared to either rGO-Ag alone or TSA alone in ovarian cells by various mechanisms including reactive oxygen species generation, mitochondrial dysfunction, and DNA damage [99]. Saranya et al. compared the anti-proliferative and apoptotic ability of CdO NPs, multiwalled carbon nanotube (c-MWCNT) NPs, and CdO/c-MWCNT nanocomposites, and CdO/c-MWCNT nanohybrids were found to have scavenging anti-cancer potential when compared with c-MWCNT NPs and CdO NP-based nanosystems [100]. The above results indicate that the coupling of carbon nanomaterials with other nanoparticles (e.g., metals and metal oxides) and drugs for combination therapy will be most effective when different nanoparticles and drugs with different action mechanisms are combined.

CDs themselves may also have anticancer activity for some gynecological cancers. Kim et al. developed some CDs with abundant hydroxyl groups, which showed excitation-dependent photoluminescence but with bright green to yellow emissions [101]. Interestingly, although the as-synthesized CDs possessed exceptional biocompatibilities and negligible toxicity for many human cell lines, they displayed antiproliferative activities against ovarian choriocarcinoma cells (JAr/Jeg-3 cell lines). The CDs derived from some drugs can retain their original antitumor activities for cancer therapy. Lu et al. synthesized drug-based CDs by microwave treatment of gallic acid (GA, one anticancer agent), which not only showed fluorescence properties but also retained the antitumor activity of GA [102]. After coupling with HeLa cell membranes as tumor targeting, the GA-based CDs had selective imaging effects and good antitumor activity on HeLa cells [103].

**Figure 7:**
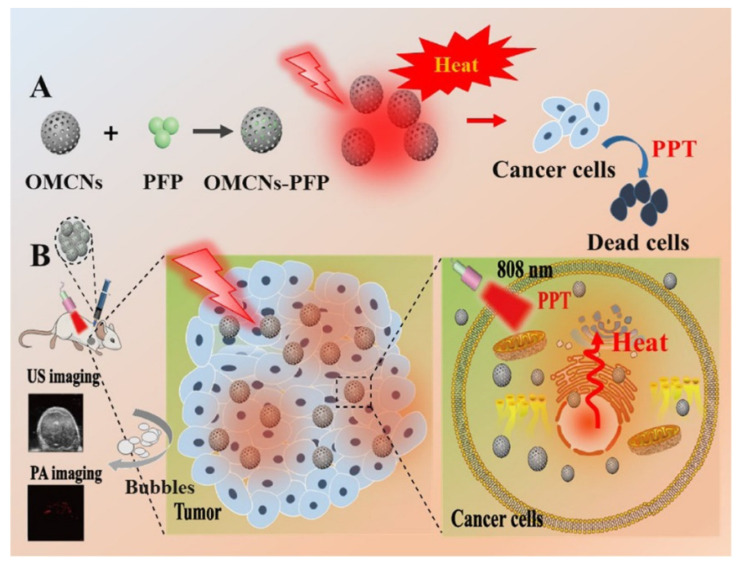
Schematic illustration of a phase-change nanotherapeutic agent based on mesoporous carbon (**A**) for ultrasound (US) imaging and photoacoustic (PA) imaging and tumor therapy (**B**) [105]. Copyright 2020, ACS.

Photothermal therapy makes use of the photothermal effect of photothermal transduction agents that can harvest the energy from light and convert the energy into heat to increase the temperature of the surrounding environment and trigger the death of cancer cells [104]. Phototherapies are the most promising therapeutic modalities offered by carbon nanomaterials. The thermal effect derived from oxidized mesoporous carbon nanoparticles (OMCNs) by NIR excitation light can not only trigger the thermal ablation of cancer cells but also promote liquid–gas phase change for gasification of perfluoropentane attached to the OMCNs, which enhances tumor ultrasound and photoacoustic imaging signals as well as photothermal therapy efficiency for HeLa cells (Figure 7) [105]. Simultaneously taking advantage of drug delivery capacity, Fang et al. reported the hyaluronic acid (HA)-modified and graphene dots (CDs)-gated hollow mesoporous carbon nanoparticles (HMCN) with a high DOX-loading capacity of 410 mg/g and excellent light-to-heat conversion property, which could realize efficient dual-responsive targeting drug delivery and synergistic chemo-photothermal therapy for CD44 receptor-overexpressed cervical carcinoma cells [106]. Moreover, the synergistic phototherapy with chemotherapy also facilitates multidrug resistance produced by chemotherapy through the multifunctional magnetic hollow and porous carbon-based nanoparticles to be overcome [91]. In addition, carbon shells as effective magnetic fluid hyperthermia can significantly reduce the magnetic agglomeration and protect the particles from oxidation, which can be used for the magnetic hyperthermia of cervical cancer [107].

**Figure 8:**
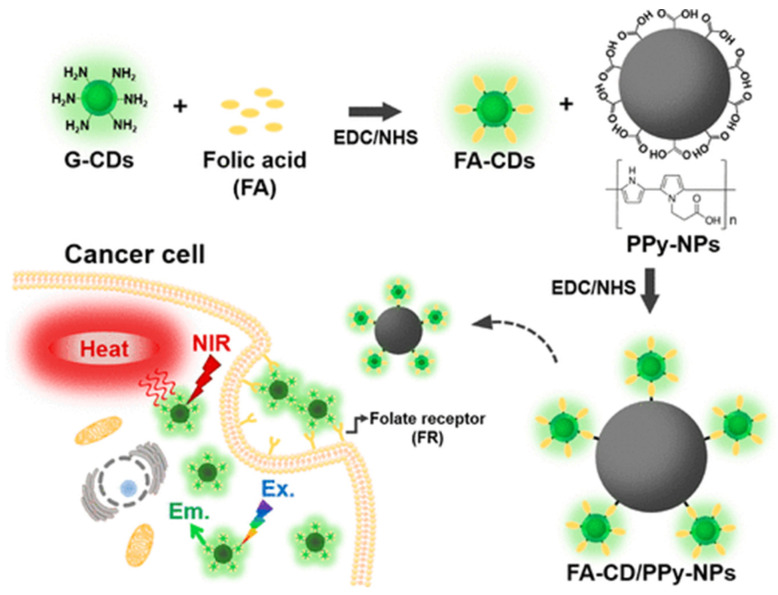
Simultaneous bioimaging and photothermal therapy by folic acid functionalized carbon dots/polypyrrole nanoparticle (FA-CD/PPy-NP) complexes [108]. Copyright 2021, ACS.

As the typical photothermal materials of CDs, coupling CDs with polymer nanoparticles always exhibits enhanced photothermal efficiency and better therapeutic performance for gynecological cancers. By combining FA functionalized CDs with Polypyrrole (PPy) nanoparticles, the FA-CDs/PPy system (Figure 8) with high photothermal conversion efficiency (η = 40.80 ± 1.54%) can kill about 87.5% of HeLa cells by NIR laser irradiation [108]. Compared with the single polydopamine (PDA) nanoparticles, the attachment of only 4 wt.% CDs for the PDA@CDs system can increase the photothermal efficiency by 30% and can kill 90% of cancer cells under 808 nm laser irradiation (50% for PDA only) [109]. The PDA@CDs system can also load 60% DOX with remarkable therapeutic performance via the synergistic effect of photothermal therapy and chemotherapy.

This recent progress demonstrates that the excellent drug loading capacity and unique photothermal properties of carbon nanomaterials are the main functions adopted to improve the chemotherapy/phototherapy efficacy of gynecological cancers. Carbon nanomaterials are always hybridized with other nanomaterials to improve the therapeutic effect. Diverse drugs and other functional reagents were also loaded on these carbon nanohybrids to enhance targeted delivery, imaging, therapy as well as biocompatibility. The synergistic chemo-photothermal therapy of gynecological cancers can also be realized by the multifunctional carbon-based nanomaterials [109]. Some elaborated carbon nanohybrids can even have the multiple abilities of imaging, drug delivery, and therapy of gynecological cancer [110]. Despite these achievements, clinical trials are still very scarce but are vitally important for the real application of carbon nanomaterials in gynecological cancers. Moreover, it is worth noting that some carbon-based nanohybrids such as rGO–Ag and CdO/c-MWCNT have good anticancer effects [98,99,100], which seem to be contradictory to many other similar carbon-based systems claimed with low toxicity and good biocompatibility [111,112,113,114,115]. The biosecurity of these carbon-based nanomedicines still should be investigated more carefully for their real application in the human body.

## 6. Biotoxicity

The toxicity of carbon-based nanomaterials in cells may vary with the administration route, the dose, the synthesis method, its physicochemical properties, and surface charge, etc. [111]. Up to now, the results about the toxicity of carbon-based nanomaterials are often contradictory. Some studies have even directly explored the carbon-based nanomaterials as anticancer drugs by virtue of their cellular damage [116], while others have claimed that they have no toxic effects with good biocompatibility [43,63]. In fact, carbon-based nanomedicines always have very complex compositions and different structures, which may result in different cytotoxicity in vitro and in vivo [117,118,119]. However, the current systematic studies of biotoxicity mainly focus on the simple model carbon such as graphenes (oxide) and carbon nanotubes, which are significantly different from the complex carbon nanohybrids with various drugs and other functional molecules [120,121,122,123]. For the biotoxicity of carbon-based nanomedicines for gynecological cancer, although most studies carried out the toxicity of carbon-based nanomedicines in cell models such as HeLa, systematic and specialized reports about their actual biotoxicity in clinical trials are still scarce. Moreover, the toxicity of the carbon-based nanomedicines on the different body organs and their metabolic pathways are still unknown, with very few research reports [124]. The biosecurity of these carbon-based nanomedicines is still greatly expected for their further clinical application in gynecological cancers.

## 7. Conclusions

In this review, we summarize the recent progress in the application of carbon nanomaterials in the theranostics of gynecologic cancers including the latest research achievements in sensing, imaging, drug delivery, therapy, and biotoxicity. In order to achieve a high diagnostic accuracy and excellent therapeutic effect, various carbon nanomaterials (e.g., graphenes, carbon nanotubes, mesoporous carbon, carbon dots, etc.) and their derivatives coupled with other nanoparticles/functional molecules were explored to increase the sensing sensitivity of the typical tumor markers, acquire better-imaged pictures, improve drug therapeutic efficacy, and realize the direct photothermal therapy for different gynecologic cancers. Carbon nanomaterials have gradually become the basic and common auxiliary units coupling with other nanoparticles and functional molecules for sensing and imaging gynecologic cancers, and some carbon-based nanomaterials such as CDs possess promising application prospects in multifunctional theranostics of gynecologic cancers including imaging, drug delivery, and photothermal therapy. Although great progress has been made from the angle of chemistry and materials science, challenges still remain to commercialize these carbon-based nanomedicines for the theranostics of gynecologic cancers in real clinical applications: (i) The carbon-based nanomaterials can be very sensitive and effective for many biosignals related with gynecologic cancers, but the fabrication of practical and convenient devices based on the fundamental research of material chemistry is still greatly desired for their clinical tests; (ii) Carbon nanoparticles suspension injection (CNSI), as the only commercialized carbon nanomaterial clinically authorized for lympha imaging, may realize the rapid clinical application for the multifunctional theranostics of gynecologic cancers when coupled with drug delivery and photothermal imaging/therapy; (iii) Although the carbon-based nanomaterials possess excellent drug loading capacity, unique photothermal properties, and thus promising application prospects for multifunctional diagnosis and therapy of gynecological cancers, the complexity of these carbon-based nanomedicines may decrease the clinical efficacy and increase the risk of biotoxicity; (iv) The biotoxicity and metabolic pathways of carbon-based nanomedicines are unknown at the current stage but will be very necessary for their further clinical application in gynecological cancers; (v) Considering the diversity of clinical responses for different patients, clinical trials and even cases analysis (e.g., CNSI) are greatly needed to acquire the really effective carbon-based nanomedicines for the theranostics of gynecological cancers. In future research, learning about the latest achievements of other nanomedicines for different cancers will also contribute to the development of effective carbon-based nanomedicines for gynecological cancers. It will be also important to make clear the difference in the therapeutic effect of carbon-based nanomedicines between gynecological cancers and other cancers to develop specific carbon-based nanomedicines for gynecologic cancers. In addition, more obstetricians and gynecologists are encouraged to participate in the research and development of new carbon-based nanomedicines and assess their application feasibility in gynecological cancers. With rapid advances in the field, carbon-based nanomedicines are expected to make a great contribution to the clinical theranostics of gynecological cancers.

## Figures and Tables

**Table 1:** The typical carbon-based nanomaterials for sensing various biosignals of gynecological cancers.

Carbon Platform Types	Tumor Types	Targeted BIOsignals	Detection Range	Detection Limit	Ref.
Graphene	Ovarian cancer	CA 125	0.025 to 250 U mL^−1^	1.9 U μU mL^−1^	[47]
Graphene	Ovarian cancer	CA 125	0.0001 to 300 U mL^−1^	0.042 μU mL^−1^	[48]
Graphene	Cervical cancer	H_2_O_2_	0.05 to 14.2 mM	2 μM	[49]
Graphene	Ovarian cancer	BRCA1 gene	10 pM to 1 mM	0.8 pM	[40]
Carbon nanotube	Ovarian cancer	CA 125	3.125 to 150 U mL^−1^	0.49 U mL^−1^	[46]
Carbon nanotube	Ovarian cancer	CA 125	1.3 to 260 U mL^−1^	2 U mL^−1^	[44]
Carbon nanotube	Ovarian cancer	CA 125	0.01–0.5 U mL^−1^ or 0.5–100 U mL^−1^	2 μU mL^−1^	[45]
Carbon nanotube	Ovarian cancer	CA 125	0.0005 to 75 U mL^−1^	6 μU mL^−1^	[33]
Carbon dots	Ovarian cancer	CA 125	0.01 to 100 U mL^−1^	9 μU mL mL^−1^	[52]
Carbon dots	Cervical cancer	H_2_O_2_	20 to 800 nM	2.4 nM	[54]
Carbon dots	Cervical cancer	pH	5.0 to 7.4	---	[55]
Carbon dots	Ovarian cancer	HE4	0.01 to 200 nM	2.3 pM	[34]
Carbon dots	Ovarian cancer	Cancer cell	2.5 × 10^3^ to 2 × 10^4^ cells mL^−1^	400 cells mL^−1^	[53]

## Data Availability

Not applicable.
